# Mutation analysis of the *p73* gene in nonastrocytic brain tumours

**DOI:** 10.1054/bjoc.2001.1855

**Published:** 2001-07

**Authors:** M E Alonso, M J Bello, P Gonzalez-Gomez, J Lomas, D Arjona, J M de Campos, M E Kusak, J L Sarasa, A Isla, J A Rey

**Affiliations:** Laboratorio de Oncogenética Molecular, Departamento de Cirugia Experimental, Unidad de Investigación, Hospital Universitario La Paz, Madrid, 28046, Spain; Departamento de Neurocirugia, Hospital del Rio Hortega, Valladolid, 47010, Spain; Departamento de Anatomia Patológica, Fundación Jiménez Diaz, Madrid, 28040, Spain; Departamento de Neurocirugia, Hospital Universitario La Paz, Madrid, 28046, Spain

**Keywords:** *p73* gene, nonastrocytic tumours, LOH 1*p36*, primary brain lymphoma

## Abstract

Loss of heterozygosity (LOH) involving the distal chromosome 1*p36*region occurs frequently in nonastrocytic brain tumours, but the tumour suppressor gene targeted by this deletion is unknown. *p73*is a novel gene that has high sequence homology and similar gene structure to the*p53* gene; it has been mapped to 1*p36*, and may thus represent a candidate for this tumour suppressor gene. To determine whether *p73*is involved in nonastrocytic brain tumour development, we analysed 65 tumour samples including 26 oligodendrogliomas, 4 ependymomas, 5 medulloblastomas, 10 meningiomas, 2 meningeal haemangiopericytomas, 2 neurofibrosarcomas, 3 primary lymphomas, 8 schwannomas and 5 metastatic tumours to the brain, for *p73* alterations. Characterization of allelic loss at 1*p36–p35* showed LOH in about 50% of cases, primarily involving oligodendroglial tumours (22 of 26 cases analysed; 85%) and meningiomas (4 of 10; 40%). PCR-SSCP and direct DNA sequencing of exons 2 to 14 of *p73* revealed a missense mutation in one primary lymphoma: a G-to-A transition, with Glu291Lys change. 8 additional cases displayed no tumour-specific alterations, as 3 distinct polymorphic changes were identified: a double polymorphic change of exon 5 was found in one ependymoma and both samples derived from an oligodendroglioma, as follows: a G-to-A transition with no change in Pro 146, and a C-to-T variation with no change in Asn 204: a delG at exon 3/+12 position was identified in 4 samples corresponding to 2 oligodendrogliomas, 1 ependymoma and 1 meningioma, and a C-to-T change at exon 2/+10 position was present in a metastatic tumour. Although both LOH at 1*p36* and *p73* sequence changes were evidenced in 4 cases, it is difficult to establish a causal role of the *p73* variations and nonastrocytic brain tumours development. © 2001 Cancer Research Campaign http://www.bjcancer.com


*British Journal of Cancer* (2001) **85**(2), 204–208
© 2001 Cancer Research Campaign
doi: 10.1054/ bjoc.2001.1855, available online at http://www.idealibrary.com on IDEAL®

http://www.bjcancer.com

### Mutation analysis of the *p73* gene in nonastrocytic brain tumours

**ME Alonso[1], MJ Bello[1], P Gonzalez-Gomez[1], J Lomas[1], D Arjona[1], JM de Campos[2], ME Kusak[2], JL Sarasa[3], A Isla[4] and JA Rey[1]**

[1]Laboratorio de Oncogenética Molecular, Unidad de Investigación, Departamento de Cirugia Experimental, Hospital Universitario La Paz, 28046 Madrid, Spain; [2]Departamento de Neurocirugia, Hospital del Rio Hortega, 47010 Valladolid, Spain; [3]Departamento de Anatomia Patológica, Fundación Jiménez Diaz, 28040 Madrid, Spain; [4]Departamento de Neurocirugia, Hospital Universitario La Paz, 28046 Madrid, Spain

**Summary** Loss of heterozygosity (LOH) involving the distal chromosome 1*p36* region occurs frequently in nonastrocytic brain tumours, but the tumour suppressor gene targeted by this deletion is unknown. *p73* is a novel gene that has high sequence homology and similar gene structure to the *p53* gene; it has been mapped to 1*p36*, and may thus represent a candidate for this tumour suppressor gene. To determine whether *p73* is involved in nonastrocytic brain tumour development, we analysed 65 tumour samples including 26 oligodendrogliomas, 4 ependymomas, 5 medulloblastomas, 10 meningiomas, 2 meningeal haemangiopericytomas, 2 neurofibrosarcomas, 3 primary lymphomas, 8 schwannomas and 5 metastatic tumours to the brain, for *p73* alterations. Characterization of allelic loss at 1*p36–p35* showed LOH in about 50% of cases, primarily involving oligodendroglial tumours (22 of 26 cases analysed; 85%) and meningiomas (4 of 10; 40%). PCR-SSCP and direct DNA sequencing of exons 2 to 14 of *p73* revealed a missense mutation in one primary lymphoma: a G-to-A transition, with Glu291Lys change. 8 additional cases displayed no tumour-specific alterations, as 3 distinct polymorphic changes were identified: a double polymorphic change of exon 5 was found in one ependymoma and both samples derived from an oligodendroglioma, as follows: a G-to-A transition with no change in Pro 146, and a C-to-T variation with no change in Asn 204: a delG at exon 3/+12 position was identified in 4 samples corresponding to 2 oligodendrogliomas, 1 ependymoma and 1 meningioma, and a C-to-T change at exon 2/+10 position was present in a metastatic tumour. Although both LOH at 1*p36* and *p73* sequence changes were evidenced in 4 cases, it is difficult to establish a causal role of the *p73* variations and nonastrocytic brain tumours development. © 2001 Cancer Research Campaign http://www.bjcancer.com

**Keywords**: *p73* gene; nonastrocytic tumours; LOH 1*p36*; primary brain lymphoma

*p53* is the most frequently mutated tumour suppressor gene identified to date (Hollstein et al, 1991), and Kaghad et al (1997) reported a novel gene, termed *p73*, that encodes a nuclear protein sharing significant sequence homology with *p53*, especially in the domains of transcriptional activation, DNA-binding and oligomerization. *p73* activates the transcription of $p21^{waf1/cip1}$, inhibits cell growth, and induces apoptosis (Jost et al, 1997); however, unlike *p53*, *p73* is not induced by exposure of cells to DNA-damaging agents such as UV irradiation (Kaghad et al, 1997).

The chromosomal localization of *p73* is proximal to marker D1S468 and distal to marker D1S47, at 1p36.33–p36.32 (Liu et al, 2000). This region is frequently deleted in a variety of human tumours (Mitelman et al, 1997). We previously reported 1p allelic deletions in about 30% of brain tumours (Bello et al, 1995a), with higher frequencies of loss found in oligodendrolgiomas and meningiomas, and a lesser degree in neurofibrosarcomas, schwannomas and primary lymphomas. Most of these tumour types display a low frequency of *p53* abnormalities, suggesting that other molecular carcinogenic pathways may participate during their progression (Ohgaki et al, 1991).

Human carcinogenesis is believed to be a multistage process involving somatic activation of protooncogenes and inactivation of tumour suppressor genes or DNA repair genes. In brain tumours, some of these genetic alterations have been outlined for astrocytic neoplasms (von Deimling et al, 1995), and recent data on molecular progression of meningioma and oligodendroglioma have emerged (Reifemberger et al, 1994; Bello et al, 1995b; Kraus et al, 1995; Simon et al, 1995; Leone et al, 1999; Smith et al, 1999). Several 1p candidate tumour suppressor genes have been analysed for inactivating mutations in oligodendrogliomas and meningiomas, including *hRAD54* and *CDKN2C* genes. There is nonetheless insufficient evidence to consider these genes as candidate tumour suppressor genes in these nonastrocytic neoplasms (Huseman et al, 1999; Mendiola et al, 1999; Bello et al, 2000b).

To evaluate possible *p73* involvement in the pathogenesis of nonastrocytic brain tumours, we analysed 65 tumour samples for mutations of the *p73* gene, and loss of heterozygosity at the 1*p36* region. Contrary to our predictions, we found that the *p73* mutation was infrequent in these tumour types.

### MATERIALS AND METHODS

### Tissue samples and DNA preparation

Normal tissues and tumour biopsies from 65 patients with nonastrocytic brain tumours were collected during surgical procedures and frozen immediately at –80˚C until use. All samples were classified by histologic examination and graded according to WHO guidelines (Kleihues et al, 1993). The group of 65 tumours

*Received 18 January 2001*
*Revised 26 March 2001*
*Accepted 27 March 2001*

*Correspondence to:* JA Rey consisted of 26 oligodendrogliomas, 4 ependymomas, 5 medulloblastomas, 10 meningiomas, 2 meningeal haemangiopericytomas, 3 primary lymphomas, 2 neurofibrosarcomas, 8 schwannomas and 5 metastatic tumours to the brain. The tumour cell content was estimated by histologic examination to be approximately 75–80%.

DNA was prepared from frozen tissues and blood samples using standard methods, as described previously (Rey et al, 1992).

### Loss of heterozygosity at 1*p36–p35*

To verify LOH at 1*p36–p35*, restriction fragment length polymorphism (RFLP) and microsatellite analyses were performed using the methods of Leone et al (1999), and the allelic constitution of the following 1p markers was determined: D1Z2, D1S80, D1S76, and D1S77, located at 1p36.33; D1S468, and D1S234, at 1p36.32; D1S199, at 1p36.12, and D1S214, at 1p35.3. The plasmids and/or oligonucleotide primers used to detect these markers have been described previously (Bello et al, 2000a), and were obtained from the American Type Culture Collection (Manassas, VA) or GENSET, SA (France). Restriction endonuclease digestion, agarose gel electrophoresis, southern blotting, $^{32}$P-labelling of DNA probes, hybridization and autoradiography were performed as described (Rey et al, 1992). Cytosine adenine repeat polymorphisms were analysed using polymerase chain reaction in standard conditions; the alleles were resolved in 6% polyacrylamide gels and then silver stained (Bender et al, 1994). Scanning densitometry was performed to determine the allelic status of markers studied by RFLP/autoradiography or PCR/SSCP/silver stain as described in detail elsewhere (Bello et al, 2000a). Loss of heterozygosity was defined as greater than 75% reduction in band intensity relative to the non-tumour control.

### SSCP analysis and direct sequencing of *p73* gene

Genomic PCR amplification of coding exons 2–14 of the *p73* gene and their splice site junction sequences were performed using the primers described by Yoshikawa et al (1999) (purchased from GENSET). PCR conditions were 35 cycles of 94˚C for 30 s, 55–68˚C for 30 s, and 72˚C for 90 s, with a final extension of 7 min at 72˚C. The PCR products were loaded onto 6–12% nondenaturing polyacrylamide gels (with or without 10% glycerol), electrophoresed and silver stained as above. Samples displaying an altered PCR-SSCP pattern were reamplified by PCR, with the same set of primers, and the PCR products were sequenced using the ABI PRISM Big Dye Terminator Cycle Sequencing Kit (Perkin Elmer, Alameda, CA). Each amplicon was sequenced bidirectionally.

### RESULTS

Allelic loss at the *p73* region (1p36.33–p36.32) could be unambiguously determined in 33 of the 65 tumour samples (50%), corresponding to 22 oligodendrogliomas, 1 ependymoma, 4 meningiomas, 1 lymphoma, 1 neurofibrosarcoma, 2 schwannomas and 2 metastatic tumours to the brain. Detailed data on the allelic constitution have been partially reported previously (Bello et al, 1995a, and unpublished data).

The genomic region from exons 2 to 14, which cover the entire coding frame of *p73*, was searched for mutations in all 65 tumours. We found a single tumour (primary lymphoma) characterised by a missense mutation, a G-to-A change at nucleotide 871 (exon 8), that is, a Glu291Lys (GAG to AAG) change (Figure 1). 2 silent mutations (or polymorphisms) were identified in both samples corresponding to grade II and grade III of an oligodendroglioma patient (case K-9). The first was a G-to-A transition at nucleotide 438 (exon 5), which does not result in aminoacid change (CCG to CCA; no change Pro 146). The second exon 5 polymorphism detected in both samples was a C-to-T change at nucleotide 612, with no change of Asn 204. Both exon 5 polymorphic variations were also detected in an ependymoma (case K-30). 5 additional tumours displayed nucleotide changes occurring in the introns. One delG at the downstream region of exon 3 (+12) position was identified in 2 oligodendrogliomas, 1 ependymoma and 1 meningioma. Finally, a C-to-T change at the exon 2(+10) position was identified in one brain metastatic lesion from a lung carcinoma. A summary of the nucleotide changes detected in all 9 tumours is shown in Table 1.

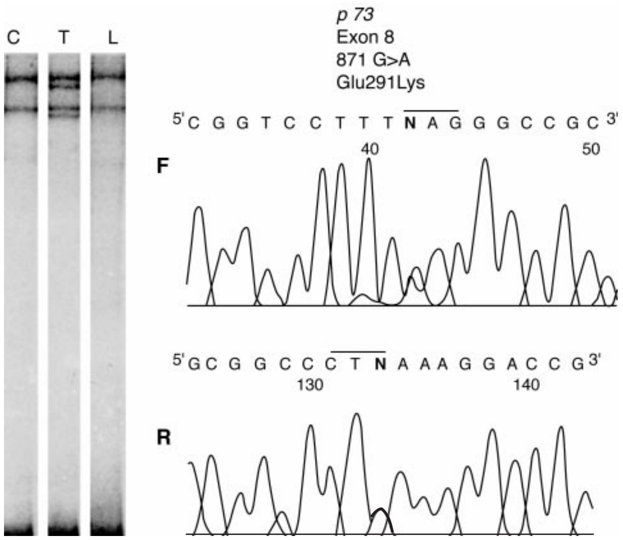

**Figure 1** Exon 8 missense mutation at position 871 of *p73* gene (G-to-A: Glu291Lys change). To the left is shown the SSCP analysis corresponding to the constitutional (L) and tumoural (T) DNAs of case K-42, and to a normal control DNA (C). Forward (F) and reverse (R) sequences corresponding to the tumour DNA show the nucleotide change

Allele loss at the 1*p36* region was identified in 4 tumours with *p73* sequence changes; these corresponded to 3 samples of oligodendroglioma and to the brain metastatic lesion from a lung carcinoma. Allelic retention was evidenced in the case with missense mutation (tumour K-42).

### DISCUSSION

Chromosomal region 1*p36* is frequently deleted in human cancer (Mitelman et al, 1997), and considered to harbour up to 3 tumour suppressor genes relevant to the carcinogenesis of a variety of neoplasms (Vergsteeg et al, 1995). We previously performed deletion mapping analysis of 1p in a broad series of 236 tumours of the nervous system, including all major histologic subtypes (Bello et al, 1995a), and determined that an average of 30% of cases displayed allelic losses at that chromosomal region. We then performed high-resolution deletion mapping analyses (composite average resolution of 4.04 cM) in nonastrocytic brain tumours and allelic imbalance at 1*p36* was identified in 28% of meningiomas and 74% of tumours with a major oligodendroglial component (Bello et al, 2000a, b). At a lower frequency, 1*p36* deletions were

© 2001 Cancer Research Campaign 

**Table 1** Mutation and polymorphisms identified in the *p73* gene in nonastrocytic brain tumours

| Sample | Tumour type | Exon/intron | Nucleotide change | AA change | LOH 1p36 |
|---|---|---|---|---|---|
| K-9 T1 | O | 5 | 438G>A/612C>T | Pro146Pro/Asn204Asn | + |
| K-9 T2 | AO | 5 | 438G>A/612C>T | Pro146Pro/Asn204Asn | + |
| K-13 | O | 3/+12 | delG | – | + |
| K-15 | O | 3/+12 | delG | – | – |
| K- 30 | E | 5 | 438G>A/612C>T | Pro146Pro/Asn204Asn | – |
| K-32 | E | 3/+12 | delG | – | – |
| K-42 | L | 8 | 871G>A | Glu291Lys | – |
| K-43 | M | 3/+12 | delG | – | – |
| K-59 | Met-LungCa | 2/+10 | C>T | – | + |

Tumour type: O = oligodendroglioma; AO = anaplastic oligodendroglioma; E = ependymoma; L = primary brain lymphoma; M = meningioma; Met-Lung Ca = lung carcinoma metastatic to the brain.

also identified in schwannomas, neurofibrosarcomas and primary lymphomas (Bello et al, 1995a, and unpublished data), and similar deletions are evidenced in the present report. These data concur with previous findings in other tumour types, prompting us to analyse molecular abnormalities of candidate genes at 1*p36*.

*p73*, a *p53*-related gene, has been located in this critical region (Kaghad et al, 1997; Liu et al, 2000) and may be the putative tumour suppressor gene involved in carcinogenesis in a variety of neoplasms, including nonastrocytic brain tumours. In this study we screened 65 nonastrocytic brain tumours for *p73* gene mutations, but identified only a single case with a missense mutation, Glu291Lys in a primary lymphoma which nevertheless retained the intact allele, shown by the retention of heterozygosity at the 1*p36* region. We also detected 8 additional cases displaying polymorphic changes, as they were also present in the corresponding constitutional DNA, but not all evidenced LOH at 1*p36*.

Taken together, our findings do not support a major role for *p73* as a tumour suppressor gene in nonastrocytic brain tumours. Similar findings were previously reported for oligodendrogliomas (Mai et al, 1998; Tsujimoto et al, 2000), as several polymorphic nucleotide variations, but no somatic mutations that caused amino acid changes were detected. High resolution deletion mapping analysis of 1p in meningioma and oligodendroglioma has demonstrated that more than one tumour suppressor gene from this genomic region might be involved. We previously performed mutational studies of the *hRAD54* gene (located at 1*p32*) in those nonastrocytic brain tumours, but no mutational changes were detected (Mendiola et al, 1999; Bello et al, 2000b). Abnormalities of the *CDKN2C* gene are likewise rarely found in oligodendrogliomas with 1p deletion (Huseman et al, 1999) and, the target gene of these highly frequent 1p deletions, characteristic of the brain neoplasms, thus remains to be identified. As far as we know, mutational analysis of the *p73* gene has been performed in several human cancers (Levrero et al, 2000), but no data are available for the other nonastrocytic brain tumour types we studied. In accordance with our findings, mutations of the gene are rarely found. In this respect, van Gele et al (2000) described the finding of a sporadic *p73* $NH_2$-terminal located missense mutation in one of 10 Merkel cell carcinomas studied. Ichimiya et al (1999) found one somatic and one germ-line mutation in a series of 140 neuroblastomas, and Han et al (1999) described a somatic missense mutation at codon 269 in one breast cancer. Peng et al (2000) identified a 5-nucleotide deletion in the DNA-binding domain of the gene in one hepatocellular carcinoma. This change resulted in a reading frame early truncation of *p73* protein, and a predicted loss of biological function. The findings in our tumour series concur with those data in that a low incidence of *p73* mutation was detected. Only one tumour sample displayed an amino acid substitution, while most SSCP variations detected corresponded to silent polymorphisms, and less frequently to changes in the intronic sequences. These findings confirm previous results suggesting that instability in the splicing of *p73* exons occurs in cancer cells, but its role is unclear (Levrero et al, 1999).

The change we have found (Glu291Lys) is located within the DNA-binding domain (DBD) of *p73*, and might represent important implications for cancer development. A high level of homology is reached in the *p73/p63/p53* DBD (63% identity) suggesting that these proteins might bind to identical DNA sequences and thus transactivate the same promoters (Yang et al, 1998; Levrero et al, 2000). Homologous Glu-Lys mutations have been described at codon 271 of *p53* in astrocytomas (Mashiyama et al, 1991), and some truncated forms of *p73/p63*, as well as changes at *p73* codon 293 might act as dominant negative factors in respect to transactivation by *p53* and *p73* alpha (Yang et al, 1998; Fillipovich et al, 2001). Accordingly, the mutation Glu291Lys we detected might inactivate *p73*, and might be of special interest regarding lymphoma development. In this respect, allelic loss at *p73* has been described in non-Hodgkin lymphomas (Herranz et al, 2000), and stimulation of a T lymphoblastoid cell line or human lymphocytes with phytohaemagglutinin has been shown to increase *p73* expression (De Laurenzi et al, 1999). Recently, Lissy et al (2000) demonstrated that *p73* is a specific mediator of T-cell receptor activation-induced cell death, and methylation changes of *p73* that might suppress expression of the gene are common in T-cell lymphomas, acute lymphoblastic leukaemia or B-cell-derived Burkitt's lymphoma (Corn et al, 1999; Kawano et al, 1999). The loss of *p73*, thus, could lead to defects in cell-cycle regulation or confer selective growth advantages.

*p73* was originally reported to be expressed monoallelically (Kaghad et al, 1997), but there have been controversial reports on the allelic expression imbalance of this gene (Nomoto et al, 1998; Takahashi et al, 1998; Kawano et al, 1999; Yokozaki et al, 2000). Whether loss of genomic imprinting is a tumorigenic mechanism for *p73*, therefore remains controversial. Although both *p53* and *p73* may function similarly, it has been suggested that *p73* is involved primarily in development (Yang et al, 2000), and is active in response to some types of DNA damage (reviewed by White and Prives, 1999). It was recently suggested that altered *p73* expression rather than a loss of function may be involved in tumorigenesis (Levrero et al, 2000).

In conclusion, the present study clearly demonstrates frequent allelic loss at 1*p36*, where the *p73* gene is located, in nonastrocytic brain tumours. We describe the finding of a *p73* missense mutation

© 2001 Cancer Research Campaign that might inactivate the gene, in a primary intracranial lymphoma, together with the identification of 3 previously reported polymorphic changes. Nevertheless, *p73* does not seem to behave as a classical 'two-hit' tumour suppressor gene in nonastrocytic brain tumours, as our study provides evidence that mutation of this gene is unlikely to play a major role in the pathogenesis of these nervous system tumours, other than brain primary lymphomas. The high incidence of 1*p36* deletions clearly provides evidence for a tumour suppressor gene located here, whose inactivation would be a critical step in promoting tumour growth in these neoplasms.

### ACKNOWLEDGEMENTS

Support for this work was provided by grant 00/0331 from FIS, Ministerio de Sanidad. MEA, is supported by a fellowship from Comunidad de Madrid.

### REFERENCES

Bello MJ, Leone PE, Nebreda P, de Campos JM, Kusak ME, Vaquero J, Sarasa JL, Garcia-Miguel P, Queizan A, Hernandez-Moneo JL, Pestaña A and Rey JA (1995a) Allelic status of chromosome 1 in neoplasms of the nervous system. *Cancer Genet Cytogenet* **83**: 160–164

Bello MJ, Leone PE, Vaquero J, de Campos JM, Kusak ME, Sarasa JL, Pestaña A and Rey JA (1995b) Allelic loss at 1p and 19q frequently occurs in association and may represent early oncogeneic events in oligodendroglial tumors. *Int J Cancer* **64**: 207–210

Bello MJ, de Campos JM, Vaquero J, Kusak ME, Sarasa JL and Rey JA (2000a) High-resolution analysis of chromosome arm 1p alterations in meningioma. *Cancer Genet Cytogenet* **120**: 30–36

Bello MJ, de Campos JM, Vaquero J, Ruiz-Barnes P, Kusak ME, Sarasa JL and Rey JA (2000b) *hRAD54* gene and 1p high-resolution deletion-mapping analyses in oligodendrogliomas. *Cancer Genet Cytogenet* **116**: 142–147

Bender B, Wiestler OD and von Deimling A (1994) A device for processing large acrylamide gels. *Biotechniques* **16**: 204–206

Corn PG, Kuerbitz SJ, van Noesel MM, Esteller M, Compitello N, Baylin SB and Herman JG (1999) Transcriptional silencing of the *p73* gene in acute lymphoblastic leukemia and Burkitt's lymphoma is associated with 5′ CpG island methylation. *Cancer Res* **59**: 3352–3356

De Laurenzi V, Catani MV, Terrinoni A, Corazzari M, Melino G, Costanzo A, Levrero M and Knight RA (1999) Additional complexity in p73: induction by mitogens in limphoid cells and identification of two new splicing variants ε and ζ. *Cell Death Differentiation* **6**: 389–390

Fillippovich I, Sorokina N, Gatei M, Haupt Y, Hobson K, Moallem E, Spring K, Mould M, McGuckin MA, Lavin MF and Khanna KK (2001) Transactivation-deficient p73alpha (p73Deltaexon2) inhibits apoptosis and competes with p53. *Oncogene* **20**: 514–522

Han S, Semba S, Abe T, Makino N, Furukawa T, Fukushige S, Takahashi H, Sakurada A, Sato M, Shilba K, Matsuno S, Nimura Y, Nakagawara A and Hossi A (1999) Infrequent somatic mutations of the p73 gene in various human cancers. *Eur J Surg Oncol* **25**: 194–198

Herranz M, Urioste M, Santos J, Martinez-Delgado JB, Rivas C, Benitez J and Fernandez-Piqueras J (2000) Allelic losses and genetic instabilities of *PTEN* and p73 in non-Hodgkin lymphomas. *Leukemia* **14**: 1325–1327

Hollstein M, Sidransky D, Vogelstein B and Harris CC (1991) *p53* mutations in human cancers. *Science* **253**: 49–53

Husemann K, Wolter M, Buschges R, Bostrom J, Sabel M and Reifemberger G (1999) Identification of two distinct deleted regions on the short arm of chromosome 1 and rare mutation of the *CDKN2C* gene from 1p32 in oligodendroglial tumors. *J Neuropathol Exp Neurol* **58**: 1041–1050

Ichimiya S, Nimura Y, Kageyama H, Takeda N, Sunahara M, Shishikura T, Nakamura Y, Sakiyama S, Seki N, Ohira M, Kaneko Y, McKeon F, Caput D and Nakagawara A (1999) p73 at chromosome 1p36 is lost in advanced stage neuroblastoma but its mutation is infrequent. *Oncogene* **18**: 1061–1066

Jost CA, Marin M and Kaelin WG (1997) p73 is a human p53-related protein that can induce apoptosis. *Nature* **389**: 191–194

Kaghad M, Bonnet H, Yang A, Creancier L, Biscan J-L, Valent A, Minty A, Chalon P, Lelias J-M, Dumont X, Ferrara P, McKeon F and Caput D (1997) Monoallelically expressed gene related to *p53* at 1p36, a region frequently deleted in neuroblastoma and other human cancers. *Cell* **90**: 809–819

Kawano S, Miller CW, Gombart AF, Bartram CR, Matsuo Y, Asou H, Sakashita A, Said J, Tatsumi E and Koeffler (1999) Loss of p73 gene expression in leukemias/lymphomas due to hypermethylation. *Blood* **94**: 1113–1120

Kleihues P, Burger PC and Scheitauer BW (1993) Histological typing of tumors of the nervous system. WHO International Histological Classification of tumors. 2nd de. Springer Verlag: Berlin

Kraus JA, Koopman J, Kaskel P, Maintz D, Brandner S, Schramm J, Louis DN, Wiestler OD and von Deimling A (1995) Shared allelic losses on chromosome 1p and 19q suggest a common origin of oligodendrogliom and oligoastrocytoma. *J Neuropathol Exp Neurol* **56**: 1098–1104

Leone PE, Bello MJ, de Campos JM, Vaquero J, Sarasa JL, Pestaña A and Rey JA (1999) *NF2* gene mutations and allelic status of 1p, 14q and 22q in sporadic meningioma. *Oncogene* **18**: 2231–2239

Levrero M, De Laurenzi V, Costanzo A, Gong J, Melino G and Wang JYJ (1999) Structure, function and regulation of p63 and p73. *Cell Death Differentiation* **6**: 1146–1153

Levrero M, De Laurenzi V, Costanzo A, Sabatini S, Gong J, Wang JYJ and Melino G (2000) The p53/p63/p73 family of transcription factors: overlapping and distinct functions. *J Cell Science* **113**: 1661–1670

Lissy NA, Davis PK, Irwin M, Kaelin WG and Dowdy SF (2000) A common E2F-1 and p73 pathway mediates cell death induced by TCR activation. *Nature* **407**: 642–645

Liu W, Mai M, Yozomizo A, Qian C, Tindall DJ, Smith DI and Thibodeau SN (2000) Differential expression and allelotyping of the p73 gene in neuroblastoma. *Int J Oncol* **16**: 181–185

Mai M, Huang H, Reed C, Qian C, Smith JS, Alderete B, Jenkins R, Smith DI and Liu W (1998) Genomic organization and mutation analysis of p73 in oligodendrogliomas with chromosome 1p-arm deletions. *Genomics* **51**: 359–363

Mashiyama S, Murakami Y, Yoshimoto T, Sekiya T and Hayashi K (1991) Detection of p53 gene mutations in human tumors by single-strand conformation polymorphism analysis of polymerase chain reaction products. *Oncogene* **6**: 1313–1318

Mendiola M, Bello MJ, Alonso J, Leone PE, Vaquero J, Sarasa JL, Kusak ME, de Campos JM, Pestaña A and Rey JA (1999) Search for mutations of the hRAD54 gene in sporadic meningioma with deletion at 1p32. *Mol Carcinogenesis* **24**: 300–304

Mitelman F, Mertens F and Johansen B (1997) A breakpoint map of recurrent chromosomal rearrangements in human neoplasia. *Nature Genetics* **15**: 417–474

Nomoto S, Haruki N, Kondo M, Konishi H, Takahashi T, Takahashi T and Takahashi T (1998) Search for mutations and examination of allelic expression imbalance of the p73 gene at 1p36.33 in human lung cancers. *Cancer Res* **58**: 1380–1383

Ohgaki H, Eibl RB, Wiestler OD, Yasargil MG, Newcomb EW and Kleihues P (1991) p53 mutations in nonastrocytic brain tumors. *Cancer Res* **51**: 6202–6205

Peng C-Y, Tsai C-L, Yeh C-T, Hung S-P, Chen M-F, Chen T-C, Chu C-M and Liaw Y-F (2000) Genetic alterations of p73 are infrequent but may occur in early stage hepatocellular carcinoma. *Anticancer Res* **20**: 1487–1492

Reifemberger J, Reifemberger G, Liu L, James CD, Wechsler W and Collins VP (1994) Molecular genetic analysis of oligodendroglial tumors shows preferential allelic deletions on 19q and 1p. *Am J Pathol* **145**: 1175–1190

Rey JA, Bello MJ, Jimenez-Lara A, Vaquero J, Kusak ME, de Campos JM, Sarasa JL and Pestaña A (1992) Loss of heterozygosity for dsital markers on 22q in human gliomas. *Int J Cancer* **51**: 703–706

Simon M, von Deimling A, Larson JJ, Wellenreuther R, Kaskel P, Waha A, Warnick RE, Tew JM and Menon AG (1995) Allelic losses on chromosome 14, 10, and 1 in atypical and malignant meningiomas: a genetic model of meningioma progression. *Cancer Res* **55**: 4696–4701

Smith JS, Alderete B, Minn Y, Borell TJ, Perry A, Mohapatra G, Hosek SM, Kimmel D, O'Fallon J, Yates A, Feuerstein BG, Burger PC, Scheithauer BW and Jenkins RB (1999) Localization of common deletion regions on 1p and 19q in human gliomas and their association with histological subtype. *Oncogene* **18**: 4144–4152

Takahashi T, Ichimiya S, Nimura Y, Watanabe M, Furusato M, Wakui S, Yatani R, Aizawa S and Nakagawara A (1998) Mutation, allelotyping, and transcription analyses of the p73 gene in prostatic carcinoma. *Cancer Res* **58**: 2076–2077

Tsujimoto T, Mochizuchi S, Iwadate Y, Namba H, Nagai M, Kawamoto T, Sunahara M, Yamahura A, Nakagawara A, Sakiyama S and Tagawa M (2000) The p73 gene is not mutated in oligodendrogliomas which frequently have a deleted region at chromosome 1p36.3. *Anticancer Res* **20**: 2495–2498

Van Gele M, Kaghad M, Leonard JH, Van Roy N, Naeyaert JM, Geerts ML, Van Belle S, Cocquyt V, Bridge J, Sciot R, De Wolf- Peeters C, De Paepe A Caput

© 2001 Cancer Research Campaign

D and Speleman F (2000) Mutation analysis of p73 and *TP53* in Merkel cell carcinoma. *Br J Cancer* **82**: 823–826
Versteeg R, Caron H, Cheng NC, van der Drift P, Slater R, Westerveld A, Voute PA, Delatre O, Laureys G, Van Roy N and Speleman F (1995) 1p36: Every subband a suppressor? *Eur J Cancer* **31A**: 538–541
von Deimling A, Louis DN and Wiestler OD (1995) Molecular pathways in the formation of gliomas. *Glia* **15**: 328–338
White E and Prives C (1999) DNA damage enables p73. *Nature* **399**: 734–735
Yang A, Kaghad M, Wang Y, Gillet E, Fleming MD, Dotsch V, Andrews NC, Caput D and McKeon F (1998) p63, a p53 homolog at 3q27–29, encodes multiple products with transactivating, death-inducing, and dominant-negative activities. *Moll Cell* **2**: 305–316
Yang A, Walker N, Bronson R, Kaghad M, Oosterwegel M, Bonnin J, Vagner C, Bonnet H, Dikkes P, Sharpe A, McKeon F and Caput D (2000) p73-deficient mice have neurological, pheromonal and inflammatory defects but lack spontaneous tumours. *Nature* **404**: 99–103
Yokozaki H, Shitara Y, Fujimoto JY, Hiyama T, Yasui W and Tahara E (1999) Alterations of p73 preferentially occur in gastric adeno carcinomas with faveolar epithelial phenotype. *Int J Cancer* **83**: 192–196
Yoshikawa H, Nagashima M, Khan MA, McMenamin MG, Hagiwara K and Harris CC (1999) Mutational analysis of p73 and *p53* in human cancer cell lines. *Oncogene* **18**: 3415–3421

 *© 2001 Cancer Research Campaign*
